# Pathways Controlling Formation and Maintenance of the Osteocyte Dendrite Network

**DOI:** 10.1007/s11914-022-00753-8

**Published:** 2022-09-10

**Authors:** Jialiang S. Wang, Marc N. Wein

**Affiliations:** 1 Endocrine Unit, Massachusetts General Hospital, Harvard Medical School, Boston, MA USA; 2 Broad Institute of Harvard and MIT, Cambridge, MA USA; 3 Harvard Stem Cell Institute, Cambridge, MA USA

**Keywords:** Dendrite formation, Osteocyte maturation, Skeletal disease, Osteocyte-neuron similarity

## Abstract

**Purpose of Review:**

The purpose of this review is to discuss the molecular mechanisms involved in osteocyte dendrite formation, summarize the similarities between osteocytic and neuronal projections, and highlight the importance of osteocyte dendrite maintenance in human skeletal disease.

**Recent Findings:**

It is suggested that there is a causal relationship between the loss of osteocyte dendrites and the increased osteocyte apoptosis during conditions including aging, microdamage, and skeletal disease. A few mechanisms are proposed to control dendrite formation and outgrowth, such as via the regulation of actin polymerization dynamics.

**Summary:**

This review addresses the impact of osteocyte dendrites in bone health and disease. Recent advances in multi-omics, in vivo and in vitro models, and microscopy-based imaging have provided novel approaches to reveal the underlying mechanisms that regulate dendrite development. Future therapeutic approaches are needed to target the process of osteocyte dendrite formation.

## Introduction

Bone-forming osteoblasts can undergo one of at least three fates: death by apoptosis, formation of bone lining cells, and differentiation into osteocytes. Osteocytes are the most abundant and longest-lived cells in bone. Surrounded by mineralized matrix, osteocytes possess an elaborate network of dendrite-like connections that are used for mechanosensing and inter-cellular communication. The mechanisms of how some osteoblasts differentiate into osteocytes remain incompletely understood. Osteoblasts that will become osteocytes are first surrounded by the unmineralized collagenous matrix they have produced (osteoid); next, developing osteocytes initiate dendrite formation prior to matrix mineralization [1, 2]. Following the deposition of calcium and phosphate along collagen fibrils, mature osteocytes are eventually formed by the integration of new dendrites into the existing osteocyte dendrite network. Osteoblasts are cuboidal cells with abundant rough endoplasmic reticulum (ER); in contrast, osteocytes possess cigar-shaped nuclei, scant ER, and large numbers of long, branching dendrites. In addition to the dramatic cellular morphological transition associated with osteocyte maturation, osteocytes acquire distinct and novel functions to control bone strength compared to osteoblasts, including (1) regulating bone remodeling by producing paracrine-acting factors, (2) mechanosensing, and (3) maintaining mineral homeostasis [2–5]. While the characteristics and functions of the lacunar-canalicular network (LCN) have been recently reviewed [6, 7•], the specific role of dendrites and dendrite-dendrite connectivity has received less attention. In this review, we will summarize emerging knowledge on the functions of osteocyte dendrites, the pathways that control their development and maintenance, and the implications of dendritic functions in pathological conditions and human skeletal disease.

## Dendrite Morphology and Microstructure

Osteocytes are the only cells whose shape is preserved in fossils through lacunae and canaliculi. Pawlicki first reported the morphology of osteocytes and their dendritic processes in Late Cretaceous dinosaur bones [8]. The osteocyte cell body is enclosed within the lacuna and the osteocyte dendrites pass through the matrix through channels called canaliculi [7•, 9]. Osteocyte dendrites make direct contacts and exhibit periodic, fibrous connections to canalicular walls through so-called tethers or dendritic spines [10]. The heparan sulfate proteoglycan protein perlecan was reported as the major component of the “tethering” structure and is essential for the integrity of the osteocyte lacunar-canalicular network (LCN) [11, 12]. In addition to “tethers,” the “collagen hillock” is another collagen matrix projection structure that directly links the extracellular matrix (ECM) to osteocyte dendrites [13]. Integrin-mediated focal adhesions (FAs) form focal attachments and connect the ECM to cell membranes within “collagen hillocks” and serve the important role of delivering external signals to the cytoskeleton [13, 14].

## Osteocyte Dendrites and Their Functions

**Fig. 1:**
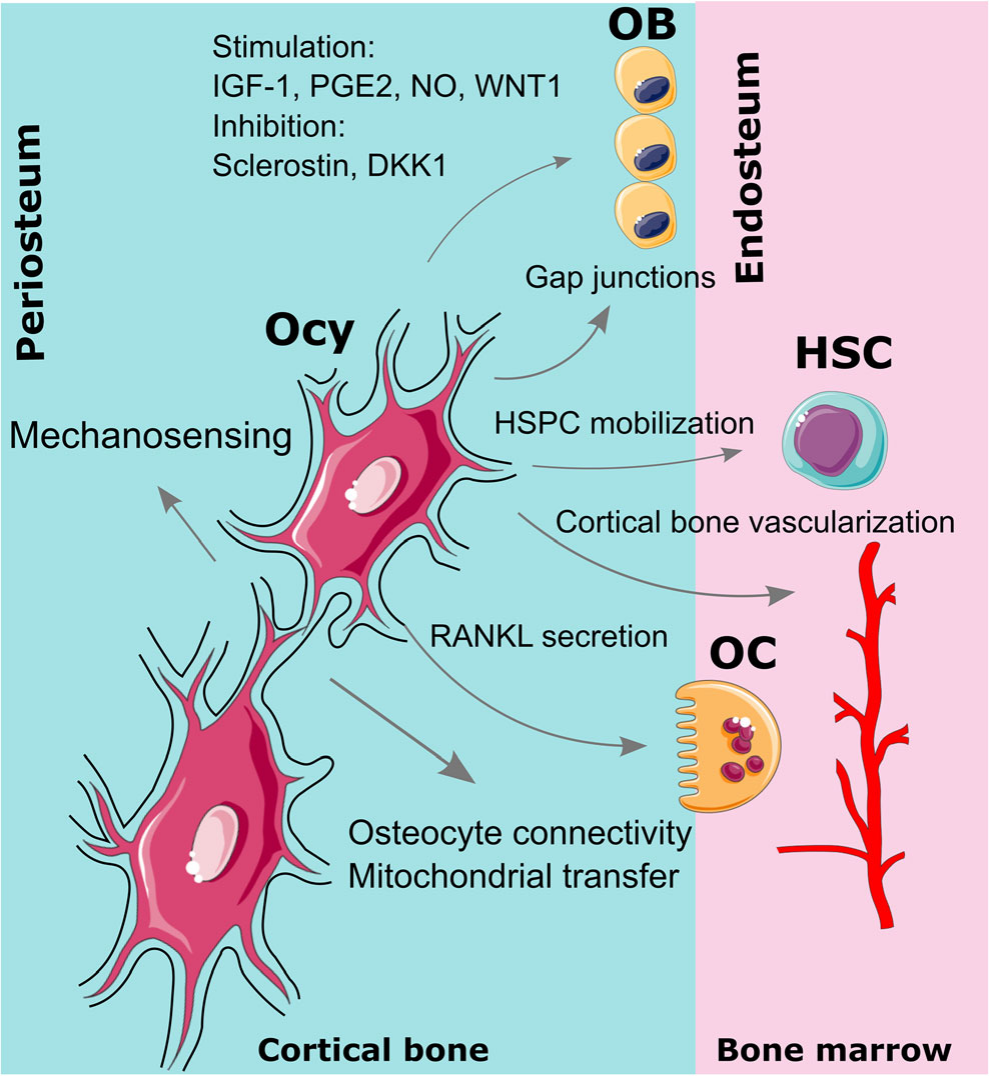
Schematic of osteocyte dendrite function. Osteocytes are the major cell type that responds to mechanical stimulation and transduces the mechanical cues into biochemical signals. Dendrites are more likely to be involved in mechano-transduction compared to osteocyte cell bodies. Osteocyte dendrites are required to maintain the lacunar-canalicular network during bone remodeling, aging, and microdamage. In addition to the osteocyte-osteocyte connectivity, osteocyte processes can regulate osteoblasts via direct contact of gap junctions, stimulate osteoclasts by triggering RANKL expression in dendrites, and control hematopoietic stem/progenitor cell (HSPC) mobilization. Ocy: osteocyte; OB: osteoblast; OC: osteoclast; HSC: hematopoietic stem cell. Part of the figure were drawn by using pictures from Servier Medical Art. Servier Medical Art by Servier is licensed under a Creative Commons Attribution 3.0 Unported License (https://creativecommons.org/licenses/by/3.0/)

The overall purpose of this review is to define the functions of osteocyte dendrites and discuss pathways responsible for their development and maintenance. Prior to discussing recent advances in osteocyte dendrite development, first we will briefly review the functions of osteocyte projections (Fig. 1): (1) cell-intrinsic roles in mechano-transduction, (2) homotypic communication with other osteocytes, and (3) heterotypic communication with other cells in the local bone microenvironment. After establishing these functions of osteocyte dendrites, we then will highlight strategies used by osteoblasts to promote the formation of these projections and their subsequent long-term maintenance.

### Mechanosensing

Osteocytes have been suggested to be the major cell type that responds to mechanical stimulation and transduces the mechanical cues to the skeleton into biochemical signals [15]. The role of osteocytes in mechano-transduction was reviewed previously [7•]; we will elaborate on how osteocyte dendrites are involved in the process. Dendrites are more likely to be involved in mechano-transduction compared to osteocyte cell bodies [16]. Burra et al. generated a transwell filter system to culture osteocytic MLO-Y4 cells, on which osteocyte cell bodies and dendrites are differentiated. The disruption of the glycocalyx on the dendrite alone is able to decrease the dendrite’s ability to open hemichannels (see below) and transmit signals to the cell body [16]. The Schaffler laboratory utilized an elegant local fluid flow stimulation approach to investigate the mechano-transducing role of dendrites and the relationship between dendrites and cell bodies in response to mechanical cues [17, 18]. Osteocytes utilize dendritic processes to receive mechanical signals, transduce these signals through major cytoskeletal components, and regulate the expression of downstream target genes.

Integrins connect the ECM to the cytoskeleton via focal adhesion (FA)–associated proteins. Integrin β3 associates with αV and is expressed in osteocyte dendrites [13, 18]. One study examined the role of integrin β3 during early corticalization and integrin β3-null mice have significantly reduced femoral length and decreased cortical thickness [19]. Chromatin immunoprecipitation (ChIP) assay performed in the osteoblastic MC3T3-E1 cells showed that the transcription factor Sp7 (see below) binds to the promoter of the *Itgb3* gene (encodes integrin β3) [19]. These findings suggest that integrin β3 acts downstream of Sp7 in regulation of corticalization for longitudinal bone growth. Since both Sp7 and integrin β3 are expressed in osteocytes, more studies are needed to explore the specific role of Sp7/Itgb3 axis in osteocyte differentiation and mechanosensing.

Gap junctions (GJs) are transmembrane channels formed on the surfaces of adjacent cells [20]. Cx43 is the most highly expressed GJs in bone. Work from the Plotkin and Bellido laboratories showed that mice lacking Cx43 in osteoblasts and osteocytes (*Oc-Cre; Cx43* ^*fl/−*^) had increased osteocytic apoptosis in cortical bone [21]. TEM images taken from the femoral midshaft of mutant mice revealed an increased number of empty lacunae and loss of dendrites in apoptotic osteocytes. In contrast, overexpression of Cx43 in osteocytes attenuated cortical bone changes during aging by preserving osteocyte viability and eventually increasing resistance to damage [22]. MLO-Y4 cells exposed to fluid flow shear stress (FFSS) increased the length of the osteocyte dendrites with redistribution of Cx43 from nucleus proximal to punctate spots in osteocyte dendrites [23]. Together, these observations suggest that Cx43 regulates osteocyte apoptosis probably via the interconnected structure of dendrites and plays an important role in bone homeostasis in response to mechanical stimulation.

The osteocyte gene *Pdpn*, which encodes E11, is increased by FFSS in vivo and in vitro, and contributes to dynamic changes in dendrite elongation seen upon mechanical stimulation [24]. The E11 glycoprotein is highly expressed in the dendrites of osteoid osteocytes and regulates dendrite initiation and elongation [25]. One recent immunocytochemical examination of the early osteoblast-to-osteocyte transition suggested that the interaction between E11 and CD44 on the cell surface, followed by ezrin phosphorylation and actin filament reorganization, may be involved in the osteoblast differentiation to osteocytes during bone remodeling [26].

### Maintaining Osteocyte Connectivity During Aging

Optimal osteocyte connectivity is thought to be needed for osteocytes to survive for long periods of time (years in the case of human osteocytes) embedded deep in mineralized bone tissue. Several studies have shown that there is a loss of osteocyte dendrites with aging. Significantly reduced dendrite number was identified in both aged human (females) and mice [27, 28]. Recent studies from the Dallas laboratory also revealed reduced canalicular and dendrite numbers in aging C57BL/6 mice [29]. There are many canaliculi that are not occupied by dendrites in aged mice, so-called empty canaliculi. Moreover, the tethering elements that connect osteocyte dendrites and canalicular walls are reduced in aged animals compared to young mice. While the molecular mechanisms used by osteocyte dendrites to protect cells from metabolic insults remain incompletely understood, it is suggested that autophagy plays an important role. Suppression of autophagy in osteocytes (*Dmp1-Cre; Atg7* ^*f/f*^, *Atg7* encodes an E1-like activating enzyme) caused low bone mass [30]. Deletion of *Atg7* with *Osx1-Cre* leads to reduced osteocyte dendrites [31]. Perhaps reduced dendrite number in *Osx1-Cre; Atg7* ^*f/f*^ mice further inhibits osteocyte maturation by disrupting the osteocyte-osteocyte connectivity, which suggests the importance of early cell dendrite initiation and embedding.

### Mitochondrial Transfer

As the major energy source for eukaryotic cells, mitochondria play a critical role in maintaining tissue homeostasis. At present, the relative role of ATP generation via glycolysis versus oxidative phosphorylation in osteocytes remains an interesting and open question [32], especially since osteocytes reside in a relatively hypoxic environment. Osteocytes may use dendritic projections to exchange mitochondria and thus preserve metabolic capacity. In addition to intra-cellular movement, inter-cellular mitochondrial transfer was first revealed between mesenchymal stem cells (MSCs) to somatic cells with mitochondrial dysfunction [33]. Both mouse primary osteocytes and MLO-Y4 cells transfer mitochondria to adjacent stressed osteocytes. This transfer takes place within osteocyte dendrites and relies on the contact between endoplasmic reticulum (ER) and mitochondria [34••]. With aging, the distribution of mitochondria in dendrites significantly reduces. Therefore, inter-cellular mitochondrial transport is required for the maintenance of the osteocyte dendritic network in aging. Further study of this interesting aspect of osteocyte biology will require identification of factors used by osteocytes to transfer mitochondria across cells.

### Regulation of Osteoblasts and Osteo-Progenitor Cells

Several paracrine signals released by osteocytes have regulatory effects on osteoblast activities (reviewed in [5]). The osteocyte-produced paracrine factor sclerostin may also regulate osteoblast progenitors [35] and osteoclasts (reviewed in [4], and see below). In addition to regulating osteoblast activities by secreted factors, osteocytes can directly regulate osteoblasts via gap junctions. In vitro co-culture of osteoblasts and osteocytes showed that under mechanical stimulation, osteocytes communicate with endosteal osteoblasts through dendritic processes and regulate the function of osteoblasts via gap junctions [36]. Osteocyte dendrites are also involved in the regulation of hematopoietic stem/progenitor cell (HSPC) mobilization [37]. Both “osteocyte-less (OL) mice” and klotho hypomorphic (*kl/kl*) mice models failed to induce HSPC mobilization by granulocyte colony-stimulating factor (G-CSF) when osteocytic dendrites and canaliculi were disrupted.

### Regulation of Osteoclasts

One in vitro and two in vivo studies showed that osteocytes are a major source of RANKL during normal bone remodeling [38–40]. Dendrites are likely important for osteocytes to transmit signals to osteoclasts. The subcellular trafficking of RANKL may be mediated by OPG in vitro that interacts with newly synthesized RANKL in lysosomes and then is transmitted to osteoclasts via osteocyte dendrites [41]. In aged female mice, loss of osteocyte dendrites precedes reduced osteocyte numbers and increases in osteoclasts [29]. Another study showed that apoptotic osteocytes in damaged bone regions signal neighboring, healthy osteocytes to secrete RANKL [42, 43]. In summary, under pathological conditions (e.g., aging and microdamage), dendrite defects may cause decreased osteocyte cell viability and increased apoptosis. The release of RANKL triggered by apoptotic osteocytes controls osteoclast localization, increases osteoclast activity, and leads to elevated bone resorption [44, 45]. The Gunzer laboratory recently reported direct physical contacts between osteocyte dendrites, endothelial cells via trans-cortical vessels (TCVs) that traverse cortical bone in a perpendicular orientation, and osteoclasts [46•]. This association between osteocyte dendrites and TCV-associated osteoclasts may induce osteoclast-mediated bone resorption through RANKL signaling and trigger TCV remodeling. This suggests a potential role of osteocyte dendrites in regulating cortical bone vascularization.

### Communication Between Osteocytes and Cancer Cells

Several cancers originate from bone, including osteosarcomas (osteoblast lineage) and myeloma (bone marrow), while others that arise from other sites metastasize to bone. Here we will discuss relationships between cancer cells and the osteocyte network. The osteocyte dendrite network is affected in the cancerous microenvironment [47, 48]. A stochastic agent-based model proposed by the Basanta Group predicted the implications of cancer cells to the osteocyte network [49]. The results showed that cancerous microenvironment can either stimulate or inhibit osteocyte dendrite growth. Direct intratibial injection of breast and prostate cancer cell lines led to direct contact between tumor cells and osteocytes [50]. The lacunar-canalicular network is impaired near osteosclerotic lesions, suggesting that bone metastasis can affect bone mechanosensitivity. In a mouse model of multiple myeloma (MM), direct contact between osteocyte dendrites and MM cells and up-regulated *Sost* expression was observed [51]. MM cells cause osteocyte apoptosis via activation of Notch signaling, and osteocytes enhance MM proliferation also via the Notch pathway [51]. Osteocyte conditioned medium stimulates the proliferation and invasion of several cancer cell lines (breast and prostate) [52]. On the other hand, mechanical loading modulates the effect of breast cancer cells on osteocyte mechanosensing by increasing the number of dendrites per osteocyte and the level of E11 [53]. The gap junction protein Cx43 forms a critical structure to inter-connect dendrites that participates in osteocyte/breast cancer communication [54]. It is likely that these studies represent just the “tip of the iceberg” with respect to how cancers in bone interact with osteocyte projections and the osteocyte network.

## Connections Between Osteocytic and Neuronal Projections

The total length of human osteocyte dendrites falls within the same range as the total length of nerve fibers in the human brain [9]. Osteocyte dendrites emanate from osteocyte cell bodies and establish a highly interconnected network. This complex communication network of osteocytes resembles the network of neurons in the brain [9]. We and others have demonstrated the similarities between osteocytic and neuronal transcriptional programs [55, 56••, 57••]. We used lineage-specific *Dmp1-Cre* transgenic mice crossed to *tdTomato* reporter mice and developed a digestion protocol to liberate osteo-lineage cells from bone matrix for single-cell RNA-seq. Gene Ontology analysis of the top markers identified from the mature osteocyte cluster revealed enrichment with “neuronal” terms such as cell projection organization and neuron differentiation. To further explore potential similarity between osteocytes and neurons at the transcriptomic level, we performed enrichment analysis of top mature osteocyte markers across cell types in mouse brain [58]. Osteocyte, but not osteoblast, marker genes are significantly enriched in their relative mean expression values in neurons versus other cell types in mouse brain. This finding suggests that osteocytes and neurons share developmental programs and signaling pathways.

One fundamental cell biology mechanism shared by both osteocytes and neurons involves development of elaborate cell projections. Our recent work demonstrated that the transcription factor Sp7 plays a crucial role in osteocyte dendrite formation. Sp7 is required for osteoblast lineage commitment [59]; however, its role in osteocyte development was poorly studied other than the observation that postnatal Sp7 ablation led to severe osteocyte morphology defects [60]. Therefore, we studied the role of Sp7 in mature osteoblasts and their descendants by deleting this gene using *Dmp1-Cre*. Surprisingly, late-stage Sp7 deletion led to osteocytes with nearly absent dendrite, increased apoptosis, and cortical porosity. These results prompted us to determine the cell-specific role of Sp7 in osteocyte dendrite development. We performed RNA-seq and ChIP-seq to determine osteocyte-specific Sp7 target genes, and identified a small group of neuronal-related genes including osteocrin. Osteocrin (encodes by the *Ostn* gene) promotes osteocyte dendrite formation downstream of Sp7 [57••]. Ostn overexpression rescues dendrite defects caused by Sp7 deficiency both in vitro and in vivo. Ostn was initially identified based on its expression in osteoblasts and early embedding osteocytes [61, 62]. *OSTN* mRNA is induced in primate (but not rodent) neocortical excitatory neurons upon depolarization. In primate neurons, OSTN regulates dendritic branch number and complexity, which suggests that OSTN inhibits neuron dendritic growth in response to excessive membrane depolarization [63].

Osteocytes and neurons may also use other common mechanisms to regulate cell survival and/or apoptosis. For example, Semaphorin 3A (Sema3A) is a secreted factor that suppresses axon growth and promotes neuronal dendrite formation via cGMP signaling (interestingly, osteocrin also potentiates cGMP signaling) [64]. Sema3A functions in bone by inhibiting bone resorption and increasing bone formation [65•]. Both postnatal global deletion of *Sema3A* and conditional deletion in osteoblasts and osteocytes result in osteoporotic phenotypes including reduced osteocyte number [66]. Sema3A activates the soluble guanylate cyclase (sGC)-cGMP signaling to promote osteocyte survival. At present, whether Sema3A signaling in osteocytes controls cellular morphogenesis or regulates dendrite maintenance remains unknown.

Shared signaling pathways could also contribute to the transcriptomic, morphologic, and functional similarities between osteocytes and neurons. Many studies reported that the ERK signaling pathway regulates neuron neurite outgrowth, number, and dendrite branching [67]. Using MLO-Y4 cells, Kyono et al. demonstrated that Fgf2 regulates osteocyte differentiation via an ERK/MAPK-dependent manner. Osteocytes in *Prx1-Cre; ERK1* ^*−/−*^*; ERK2* ^*fl/fl*^ mice have very low *Dmp1* expression and lack dendrites, indicating that the inactivation of ERK signaling pathways disrupts osteocyte maturation and dendrite formation [68]. Further in vitro studies showed that ERK activation regulates E11 expression downstream of Fgf2 [69]. Consistent with this notion, our work also demonstrated that osteocrin potentiates C-type natriuretic peptide (CNP) signaling in vitro by enhancing downstream ERK1/2 phosphorylation [57••].

Subcellular RNA localization is relatively well-studied in neurons where many RNAs are actively transported to neuronal projections for local protein translation [70, 71]. Active transport of RNA takes place along cytoskeletal scaffolds [72]. Subcellular RNA trafficking is often regulated by RNA regulatory elements (often located in the 3′ untranslated region of mRNAs) and RNA-binding proteins (RBPs) [73]. In neurons, RNA localization and local translation enhance signal transmission; defects in mRNA trafficking are linked to intellectual disability in patients with fragile X syndrome [74]. Testing whether mRNA localization also occurs in osteocyte dendrites and whether this mechanism is regulated by similar regulatory elements and RBPs identified in neurons may be an interesting future direction.

## Current Progress on Osteocyte Dendrite Formation

**Fig. 2:**
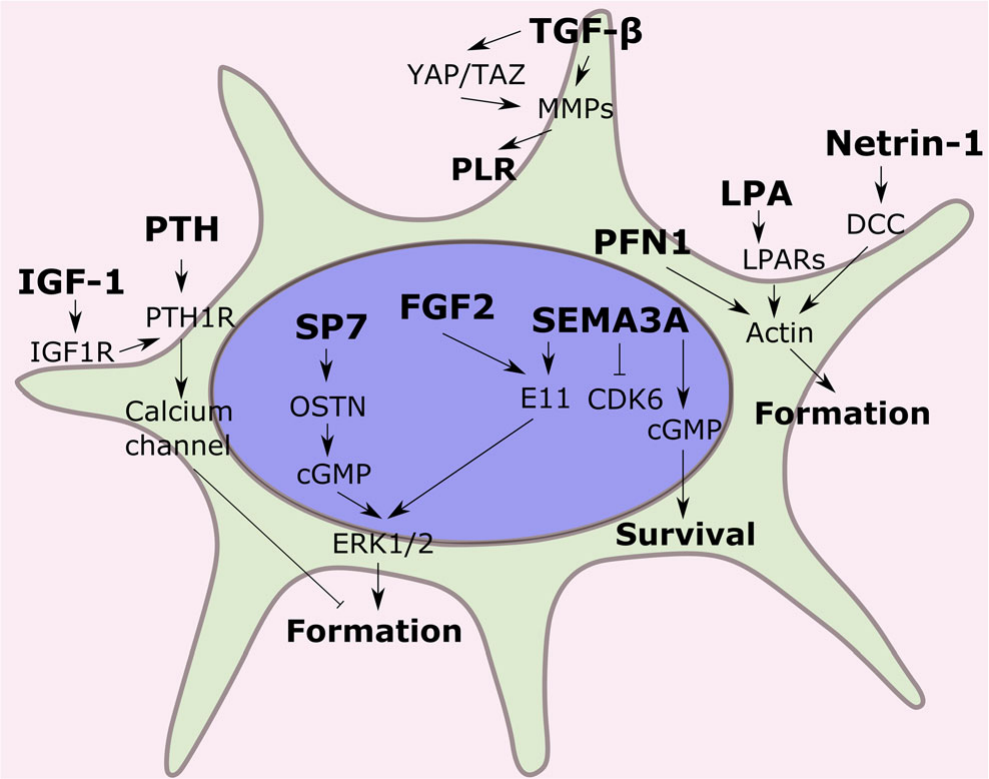
Genes and signaling pathways that are involved in osteocyte dendrite development. Several mechanisms are proposed: (1) genes (SP7, FGF2, SEMA3A) regulate osteocyte dendrite formation via cGMP levels and ERK signaling; (2) factors including lysophosphatidic acid (LPA), netrin-1, profilin1 (PFN1), and PTH/IGF-1 mediate osteocyte projection outgrowth via the actin polymerization dynamics; (3) TGF-β and YAP/TAZ pathways are essential for the perilacunar/canalicular remodeling (PLR)

While many studies reported the diverse functions of osteocyte dendrites, little is known about the mechanisms that regulate dendrite formation and osteocyte maturation. In this section, we will summarize genes and signaling pathways identified and involved in osteocyte dendrite formation (Fig. 2).

### Lysophosphatidic Acid (LPA)

The lipid growth factor lysophosphatidic acid (LPA) can induce osteocyte dendrite outgrowth [75]. Transcriptomic and proteomic analyses performed in LPA-treated MLO-Y4 cells both showed that genes and/or proteins up-regulated by LPA treatment are linked to actin microfilament dynamics, protein distribution, and membrane outgrowth [76]. This suggests that osteocyte dendrite formation is a membrane- and cytoskeleton-driven process. This is consistent with LPA-related findings in the neurons. LPA can rearrange actin cytoskeleton and microtubule in neurons [77, 78]. More studies have demonstrated the ability of LPA to induce neurite retraction and neurite branching [79, 80]. Interestingly, local LPA generation in bone has been linked to FGF-23 production in the setting of acute kidney injury [81], highlighting potential roles for LPA signaling in several aspects of osteocyte biology.

### Netrin-1

Netrins were first studied as guidance cues in neuron axon migrating [82]. Several studies indicate that netrin-1 regulates neuron axon growth through the DCC receptor [83, 84]. The Nakano laboratory recently developed a novel inkjet printing platform that contains a cross pattern of fibronectin and netrin-1 [85••]. By culturing primary osteocytes with the designed micropatterned substrates, elongation of osteocyte dendrites is selectively induced by netrin-1. This is an example of the molecular and functional similarity between osteocytic and neuronal dendrites. More in vivo studies are needed to fully understand how netrin-1/DCC signaling contributes to osteocyte dendrite elongation.

### Profilin1

Profilin1 (encoded by the *Pfn1* gene) is an actin-binding protein and is required in actin fiber polymerization [86]. Deletion of *Pfn1* increases alkaline phosphatase activity and represses dendrite formation in MLO-Y4 cells [87]. Pfn1-deficient mice (*Dmp1-Cre; Pfn1* ^*fl/fl*^) show decreased bone mineral density (BMD) and reduced trabecular bone. It is intriguing to examine whether profilin1 regulates bone mass via modulating normal osteocyte dendrite formation in the future.

### Sema3A/CDK6

Sema3A is another interesting factor that functions in osteocyte dendrite formation. As discussed above, Sema3A regulates neuron axon and dendrite growth [64]. The Yoda Group demonstrated that Sema3A promotes osteocyte dendrite elongation in vitro (MLO-Y4 cells) by down-regulation of CDK6 [88]. CDK6 is a G1 cell cycle kinase and plays an important role in tissue homeostasis and differentiation [89]. Though CDK6 down-regulation is essential for osteoblast differentiation [90], further studies are needed to elucidate the specific function of CDK6 in osteocyte maturation. Coordinating cell cycle exit may represent an important aspect of terminal osteoblast and osteocyte maturation.

### Sp7/Ostn

Our recent work reported the key role of Sp7/Ostn axis in controlling osteocyte dendrite formation [57••]. Sp7 may cooperate with distinct binding factors to regulate cell type–specific gene expression in osteocytes. Future studies are needed to understand how Sp7 orchestrates distinct gene expression programs in different cell types in bone (hypertrophic chondrocytes, early osteoprogenitors, mature osteoblasts, and osteocytes). Careful analysis of human disease–associated SP7 mutations [91–97] may provide key insights into this important question.

### PTH and IGF

It has long been understood that PTH (parathyroid hormone) can change the osteocyte cytoskeletal structure in vivo [98]. More recent work on PTH treatment of mature IDG-SW3 cell cultures alters the osteocyte projection morphology and increases osteocyte mobility [99]. Downstream analysis revealed that the change of dendrite phenotype responding to PTH is dependent on calcium signaling (increasing L-type calcium expression and decreasing T-type calcium expression). The L-type calcium channel has relatively higher expression level in osteoblasts compared to osteocytes, while the T-type calcium channel expression level is higher in osteocytes [100]. Blockage of L-type calcium channels can prevent the morphology and mobility changes caused by PTH. IGF-1 (insulin-like growth factor type 1) is expressed in osteoprogenitors, osteoblasts, and osteocytes [101]. One recent study revealed a novel PTH-IGF-1 interaction that regulates osteocyte dendrite outgrowth [102••]. IGF1R directly phosphorylates the PTH receptor (PTH1R) at tyrosine 494 in the receptor’s C-terminal tail. This specific phosphorylation targets PTH1R to the ends of actin filaments, simulates actin polymerization, and further increases osteocyte dendrite outgrowth. It remains unknown how the phosphorylated PTH1R migrates from cell membrane to the dendrite cytoskeleton.

### TGF-ß and YAP/TAZ

Perilacunar/canalicular remodeling (PLR) is a homeostatic mechanism that maintains the lacunar-canalicular network (LCN) [103]. Many factors play the crucial role in PLR, including matrix metalloproteinases (e.g., Mmp13) and cathepsin K (Ctsk) [104, 105]. Mmp13-deficient mice (*Mmp13*^*−/−*^) exhibit collagen organization and mineralization defects, and a disrupted lacunar-canalicular network, indicating that Mmp13 is essential for osteocyte perilacunar remodeling [104]. The Alliston Group described the regulatory role of the TGF-β (transforming growth factor beta) signaling pathway in osteocyte PLR [106]. Pharmacologic inhibition of TGF-β in mice results in significant reduction of canalicular length and decreased expression of PLR enzymes (Mmps, Ctsk, and Acp5). Ablation of TGF-β receptor II in osteocytes (*Dmp1-Cre; TβRII* ^*f/f*^) causes severe deterioration of the osteocyte canalicular network. TGF-β signaling interacts with YAP/TAZ signaling in many cell types [107, 108]. To examine whether YAP/TAZ regulates osteocyte-mediated bone remodeling and PLR, YAP/TAZ were conditionally deleted in osteocytes (*Dmp1-Cre; YAP* ^*f/f*^*; TAZ* ^*f/f*^). YAP/TAZ double knockout mice have increased number of empty lacunae, increased apoptotic osteocytes, and increased canalicular length compared to wild-type littermates [109•]. Further in vitro (IDG-SW3 cells) inhibition of YAP/TAZ transcriptional activity abrogates the expression of TGF-β-induced genes (*Ctgf, Cyr61*) and PLR-related enzymes, which suggests that YAP/TAZ may act downstream of TGF-β signaling to control perilacunar/canalicular remodeling.

## Dendrites and Human Skeletal Disease

Though osteocytes are buried deeply in mineralized bone matrix, the LCN provides a structural foundation for these cells to communicate and connect. Patients with skeletal disease have disrupted bone remodeling and mineral homeostasis. The molecular mechanism of how osteocyte dendrites regulate bone development and remodeling under different bone diseases remains under-studied. We will summarize the effect and importance of osteocyte dendrites in skeletal disease in the following section (Table 1).

**Table 1:** Major human skeletal disease with osteocyte dendrite defects

Disease	Dendrite defects	References
*Osteoporosis*	Decreased osteocyte connectivity, disrupted dendrite orientation, and higher dendrite tortuosity in osteoporosis patients Larger lacunar-canalicular porosity and increased effective canalicular size in ovariectomized (OVX, mimic postmenopausal osteoporosis) rats	[112, 116]
*Osteoarthritis (OA)*	Deformed osteocytes with fewer and disorganized dendrites in the subchondral bone of OA patients Decreased osteocyte viability and reduced dendrite connectivity in the femoral neck of OA patients	[112, 119, 121]
*Osteogenesis imperfecta (OI)*	Defective dendrites including reduced dendrite number and length in OI patients homologous of the *SP7 R316C* variant Defective dendritic processes in *Bmp1* and *Tll1* double knockout mice	[57••, 128]
*Glucocorticoids*	Degeneration of the lacunar-canalicular network, including loss of dendrites and rearrangement of cytoskeleton during in vitro and ex vivo culture	[136]

### Osteoporosis

Osteoporosis is a common skeletal disease characterized by decreased bone mineral density (BMD) and increased fracture risk and affects more than 200 million people worldwide. Critical osteocyte-derived factors have been reported by GWAS to show strong genetic association to BMD and fracture risk, including RANKL and SOST [110, 111]. Osteoporosis patients have decreased osteocyte connectivity, disrupted dendrite orientation (not oriented in the direction of blood supply), and higher dendrite tortuosity when compared to healthy controls [112]. At present, the genetic basis underlying osteocyte dendrite defects in osteoporosis remains unknown. Since the “osteocyte transcriptome” is enriched in genes linked to BMD variation [56••, 57••, 113], it is possible that specific genetic variants predispose certain individuals to accelerated deterioration of the osteocyte network over time. Future study is needed to better define how osteocyte-expressed BMD-associated genes control cellular morphology and dendrite homeostasis in the setting of skeletal disease. As an aging-related disease, osteoporosis results in fragility fractures in both male and female populations [114]. The ovariectomized (OVX) rat is commonly used as the model for postmenopausal osteoporosis [115]. OVX rats (estrogen deficiency) have altered lacunar-canalicular microenvironment including the larger lacunar-canalicular porosity and increased effective canalicular size [116].

### Osteoarthritis

Osteoarthritis (OA) is one of the most prevalent skeletal diseases and is characterized by articular cartilage degeneration, remodeling of the underlying bone, and inflammation of the synovium [117]. Subchondral bone (SCB) is considered the first region where the earliest change in osteoarthritic joints occurs and further triggers the degeneration of articular cartilage [118]. OA patients have deformed osteocytes in SCB with fewer and disorganized dendrites, disrupted sclerostin expression, and increased dentin matrix protein 1 expression [119, 120]. Another study also showed that the femoral neck of OA patients exhibits decreased osteocyte viability and reduced osteocytic dendrite connectivity [112]. As the factor regulating dendrite initiation and elongation [25], E11 is enriched in the sclerotic lesions of OA patients [121, 122]. Elevated E11 level in early osteocyte dendrite forming phase may inhibit bone resorption in OA femoral head bone. Future studies are needed to precisely define “cause and effect” regarding changes in osteocyte biology in subchondral bone in the setting of OA progression.

### Osteogenesis Imperfecta

Osteogenesis imperfecta (OI) is a relatively common skeletal dysplasia, affecting between 15,000 and 20,000 patients in the USA [123]. Though the majority of OI cases are caused by variants in *COL1A1* and *COL1A2* genes, a large number of genes, including genes crucial for osteoblast and osteocyte function, have been identified to cause skeletal fragility and a phenotype similar to “classic” (collagen-mutated) OI [124, 125]. For example, rare *SP7* mutations cause recessive forms of OI [94, 95]. Homozygous *SP7 R316C* patients are characterized by short stature, recurrent fractures, and high cortical porosity. Recent studies from our laboratory demonstrated reduced osteocyte dendrite length and number in homozygous *SP7 R316C* patients compared to age-matched controls [57••]. *BMP1* is another candidate gene identified in recessive OI by exome sequencing [126, 127]. Postnatal deletion of *Bmp1* and *Tll1* (encodes Tolloid Like 1) in mice showed defective dendritic processes [128]. In vivo transcriptomic analysis performed in OI mouse models (*CrtapKO* and *oim/oim*) identified differentially expressed genes that are significantly enriched in “Cell Projection” and “Neuron Projection,” which suggests that the connectivity of osteocyte dendritic network may be affected in OI [129]. Taken together, these observations indicate that osteocyte dendrite defects may be a common feature across multiple forms of OI, and suggest that targeted interventions to restore dendrite viability may represent a novel treatment strategy for this serious and debilitating skeletal disease.

### Glucocorticoids

Glucocorticoids (GCs) are widely used as anti-inflammatory drugs to treat inflammatory diseases including rheumatoid arthritis, multiple sclerosis, asthma, lupus, and inflammatory bowel disease [130]. However, long-term GC treatment is associated with skeletal side effects including bone loss, fracture, osteoporosis, and osteonecrosis [131]. Both in vivo and in vitro studies showed that excess GCs induce osteocytes apoptosis [132–135]. In glucocorticoid-treated mice, perilacunar remodeling is suppressed due to inhibition of matrix metalloprotease expression, which eventually causes degeneration of the osteocyte lacunar-canalicular network [135]. It is suggested that GCs regulate osteocytes in a dose-dependent manner: osteocytes undergo the autophagy pathway with lower GC dose, while high GC dose induces osteocyte apoptosis [134]. Go et al. reported that dexamethasone (Dex) inhibits Cx43 expression at sites of inter-cellular connections via dendrite tips [136]. Dex-administration during in vitro and ex vivo culture causes loss of osteocyte dendrites and cytoskeletal rearrangement due to autophagy-medicated Cx43 degradation [136]. Therefore, preserving or regenerating osteocytic dendrite network during GC excess may provide a novel direction in preventing GC-induced skeletal complications. Doing so will require a more detailed understanding of how glucocorticoids lead to osteocyte dendrite loss.

## Gaps and Limitations in Studying Osteocyte Dendrites

Current methods to visualize osteocytes and their dendrites are somewhat challenging. Silver nitrate can be used to stain osteocyte cell bodies and processes dark brown [106, 137]. Conjugated forms of phalloidin, which stains filamentous actin, can be used to visualize the osteocyte cytoskeleton coursing throughout acellular bone matrix [138]. We and others have applied phalloidin staining in cultured osteocytes to visualize dendrite development and osteoblast-to-osteocyte differentiation [57••, 139, 140]. The Dallas Group has developed multiplexed confocal imaging methods for imaging different aspects of osteocytes (DAPI: nucleus; phalloidin: cytoskeleton; mineralized matrix: alizarin red; collagen: 2nd harmonic generation) combined with fluorescence-conjugated dextran dye that permeates the lacunar-canalicular system [141]. In addition, the Dallas laboratory has also applied live cell confocal imaging to visualize the dynamics of osteocyte embedding and dendrite formation in mice co-expressing GFPtopaz-tagged collagen and Dmp1-Cre/tdTomato [142]. The combination of confocal imaging, live imaging, and streamlined methods for image quantification and analysis will provide novel insights into the formation of osteocyte dendrites and the lacunar-canalicular system.

## Conclusions

Osteocyte dendrites serve as functional structures that regulate osteocyte function and bone health. Evidence has suggested a causal relationship between the loss of osteocyte dendrites and the increased osteocyte apoptosis. Apoptotic osteocytes eventually trigger bone loss and bone fracture. An enhanced understanding of osteocyte dendrite development and maintenance will highlight new ways to regenerate these structures in skeletal disease. Novel therapeutic approaches are needed to target the processes of dendrite formation and maintenance. Osteocytes and neurons share similarities at the morphological, transcriptional, and functional levels. Osteocytes may repurpose neuronal molecular control pathways to regulate dendrite formation, cell survival, and mRNA transport to dendrites. Leveraging knowledge from neuroscience research is likely to accelerate understanding of how the osteocyte network forms and functions.
